# The psychometric properties of the ‘safety attitudes questionnaire’ in out-of-hours primary care services in the Netherlands

**DOI:** 10.1371/journal.pone.0172390

**Published:** 2017-02-16

**Authors:** Marleen Smits, Ellen Keizer, Paul Giesen, Ellen Catharina Tveter Deilkås, Dag Hofoss, Gunnar Tschudi Bondevik

**Affiliations:** 1 Radboud Scientific Center for Quality of Healthcare (IQ healthcare), Radboud Institute for Health Sciences, Radboud university medical center, Nijmegen, The Netherlands; 2 Health Services Research Unit, Akershus University Hospital, Lørenskog, Norway; 3 The Norwegian Directorate of Health, Oslo, Norway; 4 Institute of Health and Society, University of Oslo, Oslo, Norway; 5 Department of Global Public Health and Primary Care, University of Bergen, Bergen, Norway; 6 Uni Research Health, National Centre for Emergency Primary Health Care, Bergen, Norway; University of Ottawa, CANADA

## Abstract

**Background:**

The Safety Attitudes Questionnaire (SAQ) is one of the most widely used instruments to assess safety culture among healthcare providers. The ambulatory version of the SAQ (SAQ-AV) can be used in the primary care setting. Our study objective was to examine the underlying factors and psychometric properties of the Dutch translation of the SAQ-AV in out-of-hours primary care services.

**Design:**

Cross-sectional observational study using a web-survey.

**Setting:**

Sixteen out-of-hours general practitioner cooperatives and two call centers in the Netherlands.

**Participants:**

Primary healthcare providers in out-of-hours services.

**Main outcome measures:**

Item-descriptive statistics, factor loadings, Cronbach’s alpha scores, corrected item-total correlations, scale correlations.

**Results:**

The questionnaire was answered by 853 (43.2%) healthcare professionals. In the factor analyses, 784 respondents were included; mainly general practitioners (N = 470) and triage nurses (N = 189). Items were included in the analyses based on question type and results from previous studies. Five factors were drawn with reliability scores between .49 and .86 and a good construct validity. The five factors covered 27 of the 62 questionnaire items, with three to five items per factor.

**Conclusions:**

The Dutch translation of the SAQ-AV, with five factors, seems to be a reliable tool for measuring patient safety culture and guide quality improvement interventions in out-of-hours primary care services. The Dutch factor structure differed from the original SAQ-AV and other translated versions. In future studies, the questionnaire should be validated further by examining if there is a relationship between the responses on the SAQ-AV, patient experiences, and the occurrence of adverse events.

## Introduction

To improve quality and safety in healthcare, organisations have to create a positive patient safety culture. Patient safety culture is how leader and staff interaction, attitudes, routines and practices protect patients from adverse events in healthcare [1]. The phenomenon exists in groups of people working together—in natural social units like for example hospital wards and ambulatory clinics- and not in single individuals alone [2,3]. Over the last years, the focus in patient safety research has mainly been upon hospital care [4]. Most patients, however, receive their healthcare in primary care settings, particularly in countries with a strong primary care system [5]. Primary care differs from hospital care in terms of organisational structure, administrative and clinical processes and the reasons for encounter. Therefore, also patient safety culture dimensions could differ between the settings [6].

If healthcare organisations want to improve patient safety, it is important to know more about the patient safety culture. Several instruments are available to assess safety culture [7–12]. A widely used instrument to measure patient safety culture is the Safety Attitudes Questionnaire (SAQ) [13]. It can be used in different healthcare setting [14]. Measurements of safety culture, which is an aspect of the organisational culture, are referred to as climates [1]. Previous research has shown that SAQ climate scores correlate with patient outcomes [7, 15–16]. Moreover, the instrument may identify possible weaknesses in a clinical setting and this can stimulate quality improvement interventions [17,18]. From the original SAQ, a questionnaire for measuring safety culture in outpatient settings was developed, adjusted to and tested in the primary care setting [9,19]. This ambulatory version of the SAQ (SAQ-AV) was used in an international study entitled Patient Safety Culture in European Out-of-hours services (SAFE-EUR-OOH) which was led by a coordinating research group from Norway [19]. The study was a project of the European research network for out-of-hours primary health care (EurOOHnet) [20]. We translated the SAQ-AV into Dutch and adjusted it for specific application in out-of-hours primary care services, also called general practitioner (GP) cooperatives, in the Netherlands (see Table 1 for general characteristics of Dutch GP cooperatives).

**Table 1:** Features of general practitioner (GP) cooperatives in the Netherlands [21].

Theme	Feature
General	Out-of-hours primary care has been provided by large-scale general practitioner (GP) cooperatives since the year 2000
	About 120 GP cooperatives in the Netherlands
	Out-of-hours defined as daily from 5 p.m. to 8 a.m. holidays and the entire weekend
	Population of 100,000 to 500,000 patients with an average care consumption of 250/1000 inhabitants per year
	Participation of 50–250 GPs per cooperative with a mean of 4 hours on call per week
	Per shift GPs have different roles: supervising telephone triage, doing centre consultations or home visits
Location	GP cooperative usually situated in or near a hospital’s Accident and Emergency department (A&E)
	Distance of patients to GP cooperative is maximally 30 km
Accessibility	Access via a single regional telephone number (only 5–10% walk in without a call in advance)
	Telephone triage by nurses supervised by GPs: contacts are divided into telephone advice (by triage nurse or GP) (40%), GP clinic consultation (50%), or GP home visit (10%)
	Some GP cooperatives use a central call center for telephone triage
Facilities	Home visits are supported by trained drivers in identifiable fully equipped cars (e.g. oxygen, intra venous drip equipment, automated external defibrillator, medication for acute treatment)
	Information and communication technology (ICT) support, including electronic patient files, online connection to the GP car, and sometimes connection with the electronic medical record in the GP daily practice.

Patient safety is of particular importance in GP cooperatives, because of a high patient throughput, diversity of urgent clinical conditions presented, identification of medical urgency during telephone contacts, and limited knowledge of the medical history of the patient. In addition, the GPs work in shifts and have to collaborate with other healthcare providers, which increases the risk of errors caused by discontinuity in information transfer [22, 23].

After translating a questionnaire into another language and applying it in a different setting, it is important to test the validity of the questionnaire in the new context. In addition, if the psychometric properties of the Dutch version of the SAQ-AV are comparable to the original questionnaire, cross-country comparisons can be performed to gain more insight into similarities and differences in patient safety culture between countries. The aim of this study was to examine the underlying factors and psychometric properties of the Dutch translation of the SAQ-AV in GP cooperatives.

## Materials and methods

### Setting

The study was performed in a convenience sample of 16 out-of-hours GP cooperatives and two call centers in the Netherlands (see Table 1). The two call centers performed the telephone triage of all calls to seven of the 16 GP cooperatives. The GP cooperatives were spread over the East, South and West of the Netherlands and varied in size and urbanisation grade. They served a total population of 2.050.000 inhabitants and employed a total of 2015 healthcare professionals, of whom 76.2% GP’s, 15.9% triage nurses and 7.9% other personnel. Locum doctors who had worked less than five shifts during the past year, were excluded from the study beforehand.

As part of the international SAFE-EUR-OOH project, the study was also performed in Norway (coordinating country), Slovenia, Italy and Croatia. The translation and data collection procedures were equal in each country.

### Translation procedure

The SAQ-AV questionnaire was translated following modified principles adapted from Beaton et al [24]. Initially, the original English items [9] were translated into Dutch using a professional Dutch native translator. Next, an expert panel of two GPs, two triage nurses and two researchers adapted the initial translated version to the out-of-hours primary care setting in the Netherlands (for example “office” was changed into “GP cooperative” and “e.g. biopsy” into “e.g. surgical procedure”). This slightly adapted version of the questionnaire was translated back into English by a second independent professional English native translator, who was blinded to the original version. Based on this back-translated version, the expert panel made some adjustments in order to clarify misunderstandings. The prefinal Dutch version was tested in a small group of primary healthcare providers. This did not result in any further adjustments. Pre-tests showed that it took approximately 15 minutes to complete the questionnaire.

### Questionnaire

*Background variables*: Work-related information, e.g. the respondent’s profession, years of experience, number of working hours a week.

*Items on patient safety culture*: The SAQ-AV contains 62 items on patient safety culture. Respondents rate their agreement using a 5-point Likert scale: 1 = disagree strongly, 2 = disagree slightly, 3 = neutral, 4 = agree slightly, 5 = agree strongly. For all questions, “Not applicable” was included as a response category. The original SAQ-AV described six factors covering 30 of the 62 items: Teamwork climate, Safety climate, Working conditions, Job satisfaction, Perceptions of management and Stress recognition with Cronbach’s alpha scores between 0.68 and 0.86 [9].

### Data collection and procedure

The key contact persons of the GP cooperatives provided the e-mail addresses of all professionals having direct patient contact in their clinical work. In January and February 2015, the SAQ-AV was distributed by a link in an e-mail to 2015 primary healthcare providers in these 16 GP cooperatives and two call centers. In the preceding month, the contact person in each GP cooperative informed the staff about the study during work meetings, on the intranet, with posters and by email. Data were collected electronically using the program Qualtrics, whereby the participants responded anonymously. All questions were obligatory to answer. This data collection program automatically sent a reminder to those who had not responded after two weeks and after one month. After three weeks, an additional reminder was sent to the contact persons of the GP cooperatives, asking them to motivate the clinical staff to participate in the study. After the study, each of the participating GP cooperatives received a feedback report with the results of their unit, including a comparison of their results with the mean results of the total group. In this way, the healthcare providers were encouraged to focus on specific factors related to patient safety, and to discuss possible strategies for improvement within their clinical setting.

### Data screening and pre-analyses

Completeness of the data was checked, resulting in an exclusion of 69 respondents, because they had completed less than half of all safety culture items—they all prematurely ended the questionnaire. There were no variables with 65% or more answers in one category, thus no floor or ceiling effects.

We checked whether the inter-item correlations were sufficient, by an examination of the correlation matrix. Questions belonging to the same underlying dimension will correlate, as they measure the same aspect of patient safety culture. Items that do not correlate, or correlate with only a few other variables are not suitable for factor analysis. Bartlett’s test demonstrated that the inter-item correlations were sufficient: χ^2^ = 7478.3; df = 351; p < .001. We also checked whether the opposite occurred: too high correlations between the items. Ideally, every aspect of patient safety culture uniquely contributes towards the concept of patient safety culture. No correlations exceeded the boundary score of 0.7 [25].

In addition, The Kaiser-Meyer-Olkin Measure of Sampling Adequacy (KMO) was determined. This value can range from 0 to 1. A value near 1 indicates that there is hardly any spread in the correlation pattern, enabling reliable and distinctive dimensions by factor analysis. The KMO-score was 0.9; far above Kaiser’s criterion of 0.5 [26]. The pre-analyses demonstrated that the data could be used for factor analysis.

To enable future comparisons of patient safety culture in out-of-hours setting across countries, we tried to fit the factor structure of the Norwegian questionnaire responses [19] (by confirmatory factor analysis program AMOS—not reported in this article). This did not confirm that the factor structure of the Norwegian questionnaire was also present in the Dutch data. The data were therefore studied with exploratory factor analysis to check whether the items form different factors in the Dutch out-of-hours primary care setting.

### Statistical analyses

The Qualtrics file with anonymous SAQ-AV data was converted into an SPSS (Statistical Package for the Social Sciences) file for further analysis (IBM SPSS 22). The response category “Not applicable” was treated as a missing value in the data analyses (0%-2% missing values per item). Since the questionnaire contains positively as well as negatively worded items, the negatively formulated items were first recoded to make sure that a higher score always meant a more positive response. For each item, the mean and standard deviation were calculated.

We performed an exploratory factor analysis (Principal Components) with Varimax rotation. To find the most appropriate factor model, we explored the factor structures of different sets of items. Some items were permanently excluded from these exploratory factor analyses: 1) items consisting of general statements that are not necessarily applicable to the specific situation of the respondent, e.g “Truly professional personnel can leave personal problems behind when working” (10 items); 2) items for which it was unclear if a higher score meant a more positive or negative safety culture, e.g. “I have made errors that had the potential to harm patients” (two items), and 3) an item that was ambiguous according to two respondents: “Office management does not knowingly compromise the safety of patients” (one item). As an exploratory factor analysis with the remaining 49 items did not yield an interpretable model, we started with a set of items that were either present in the factors structures of Norway [19] or Slovenia (both SAFE-EUR-OOH study countries) [6]. In subsequent steps, we deleted items that did not fit in the Dutch factor structure and added items that were not present in the Norwegian or Slovenian structures, but which reasonably belonged to one of the factors in our structure, based on the items’ content.

In the exploratory factor analyses, missing values were deleted pairwise. When establishing the number of factors, the Eigen value (Eigen value>1: Kaiser’s criterion) was taken into account, beside the extent of explained variance, the shape of the scree plot and the possibility of interpreting the factors. Kaiser’s criterion is reliable in a sample of more than 250 respondents and when the average communality equals or is larger than 0.6. The shape of the scree plot gives reliable information when the sample is larger than 200 respondents [25]. The data satisfied these conditions.

The internal consistency of the factors was calculated with Cronbach’s alpha (α). If different items are supposed to measure the same concept, the internal consistency (reliability) should be greater than or equal to 0.7 [27]. For each item, the correlation between the item and the total score was calculated (corrected item-total correlations). In a reliable scale, all items should correlate with the total (r > 0.3) [25].

Finally, the construct validity was studied by calculating scale scores for every factor and subsequently calculating Pearson correlation coefficients between the scale scores. The construct validity of each factor is reflected in scale scores that are moderately related. High correlations, however, would indicate that factors measure the same concept and these factors may be combined and/or some items could be removed. For each factor, also the mean and standard deviation were calculated.

### Ethical considerations

This study was based on data regarding patient safety culture among healthcare providers. Participation was voluntary. All participants received written information about the purpose of the study, and that the data were collected anonymously and treated in confidence. The Ethical Research Committee concluded that this study does not fall within the remit of the Dutch Medical Research Involving Human Subjects Act [Wet Mensgebonden Onderzoek] (file number 2014–299).

## Results

### Respondents

**Fig 1:**
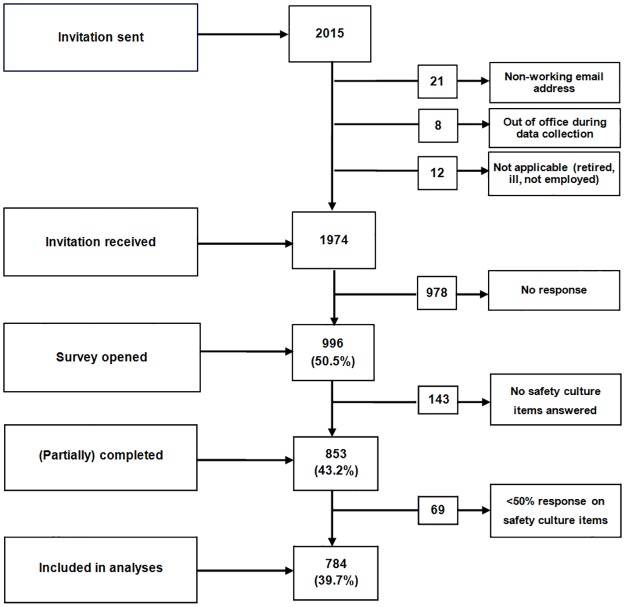
Flowchart of the response to the SAQ-AV.

Of the 2015 employed healthcare professionals, 1974 correctly received an invitation to complete the questionnaire on a working email address, of which 853 (43.2%) answered the questionnaire (Fig 1).

Analyses were performed on the 784 respondents who answered more than half of all safety culture items. Of the respondents, 526 (68.6%) were female and 233 (30.4%) were aged between 41 and 50 years. Most of the respondents were GPs (61.2%; N = 470) or triage nurses (24.6%; N = 189). The majority had been working at the current GP cooperative for 11 to 20 years (36.0%; N = 276) (Table 2).

**Table 2:** Characteristics of the respondents.

Characteristic	N (%)
**Gender (N = 767)**	
Female	526 (68.8)
**Age (N = 767)**	
≤ 30 y	95 (12.4)
31–40 y	196 (25.6)
41–50 y	233 (30.4)
51–60 y	194 (25.3)
≥ 61 y	49 (6.4)
**Occupation (N = 768)**	
GP	470 (61.2)
Triage nurse	189 (24.6)
Driver	39 (5.1)
Specialised nurse	24 (3.1)
Administrator	21 (2.7)
Manager	16 (2.1)
Medical student	9 (1.2)
Working experience* **(N = 767)**	
≤ 2 y	136 (17.7)
3–5 y	168 (21.9)
6–10 y	187 (24.4)
11–20 y	276 (36.0)

* At current GP cooperative

### Exploratory factor analysis

Five factors were drawn by exploratory factor analysis, covering 27 items of the questionnaire and jointly explaining 52.4% of the variance in the responses: Perceptions of management, Job satisfaction, Teamwork climate, Safety climate and Communication openness. The factors consisted of three to nine items. Cronbach’s alpha scores varied between 0.49 for Communication openness and 0.86 for Perceptions of management. Table 3 shows the mean scores, standard deviations, factor loadings and corrected item-total correlations of each item in the factor structure. The mean scores of the items varied between 3.05 (for item 39r ‘I am frequently unable to express disagreement with staff physicians/intensivists in this office’) and 4.44 (for item 52r ‘I feel frustrated by my job’). For one item (39r), the corrected item-total correlation was below 0.3 (r = 0.235).

**Table 3:** Mean scores, standard deviations, factor loadings and corrected item-total correlations of the 27 items in the factor structure and Cronbach’s alpha for the five factors.

Nr.	Item	Mean	SD	F1	F2	F3	F4	F5	CITC
**Perceptions of management**—*Cronbach’s α = 0*.*86*									
9	Senior management of this office is doing a good job	3.85	.959	.788					.722
10	The management of this office supports my daily efforts	3.74	1.04	.748					.647
19	Decision making in this office utilizes input from relevant personnel	3.72	.965	.634					.590
5	Medical errors are handled appropriately in this office	4.25	.841	.614					.605
22	This office deals constructively with problem personnel	3.61	.941	.506			.*443*		.510
30	Disagreements in this office are resolved appropriately (i.e. not *who* is right but *what* is best for the patient)	3.85	.917	.534					.609
6	This office does a good job of training new personnel	4.13	.882	.589					.586
3	Nurse input is well received in this office	4.30	.792	.512					.552
26	I am provided with adequate, timely information about events in the office that might affect my work	3.97	.892	.427					.512
**Job satisfaction**—*Cronbach’s α = 0*.*81*									
15	This office is a good place to work	4.34	.845		.795				.752
2	I like my job	4.43	.809		.841				.621
52r	I feel frustrated by my job	4.44	.852		.599				.435
29	I am proud to work at this office	4.07	.910	.*409*	.592				.638
8	Working in this office is like being part of a large family	3.53	1.08	.*442*	.545				.567
**Teamwork climate***—Cronbach’s α = 0*.*77*									
45	Attending physicians/primary care providers in this office are doing a good job	4.26	.640			.707			.449
35	It is easy for personnel in this office to ask questions when there is something that they do not understand	4.31	.735			.595			.548
38	The physicians and nurses here work together as a well-coordinated team	4.16	.805			.615			.620
34	I have the support I need from other personnel to care for patients	4.07	.838			.541			.549
42	Trainees in my discipline are adequately supervised	4.25	.827			.537			.418
50	Important issues are well communicated at shift changes	4.00	.883			.501			.485
**Safety climate**—*Cronbach’s α = 0*.*62*									
37	During emergencies, I can predict what other personnel are going to do next	3.33	.932				.696		.330
20	I am encouraged by my colleagues to report any patient safety concerns I may have	3.71	.982				.593		.422
21	The culture in this office makes it easy to learn from the errors of others	3.91	.894				.485		.451
28	I know the proper channels to direct questions regarding patient safety in this office	3.93	.990				.468		.408
**Communication openness**—*Cronbach’s α = 0*.*49*									
24r	In the office, it is difficult to speak up if I perceive a problem with patient care	3.61	1.22					.709	.382
12r	In this office, it is difficult to discuss errors	3.71	1.13					.662	.311
39r	I am frequently unable to express disagreement with staff physicians/intensivists in this office	3.05	1.27					.590	.235

Notes: Factor loadings >0.40 are shown.

Factor loadings in italics indicate that this was not the preferred option.

The letter ‘r’ in a code means that it concerns an item in negative wording, which was reverse coded.

No item correlated more strongly with other factors than with its own factor.

SD = Standard Deviation

CITC = Corrected Item-Total Correlation

### Construct validity

For each of the five factors, scale scores were calculated by obtaining the mean of the item scores within one factor for every respondent. Next, correlations between the scale scores were calculated. Table 4 shows the mean scale scores with standard deviations, and the correlations between the factors.

**Table 4:** Mean scores, standard deviations and intercorrelations of the five factors.

Nr	Factor title	Mean	SD	F1	F2	F3	F4
F1	Perceptions of management	3.94	.633				
F2	Job satisfaction	4.16	.682	.535			
F3	Teamwork climate	4.17	.545	.630	.569		
F4	Safety climate	3.72	.653	.630	.473	.559	
F5	Communication openness	3.46	.852	.339	.289	.320	.259

Note: All correlations are significant at p< 0.01.

The highest correlations were those between Perceptions of management and Teamwork climate (r = 0.63) and between Perceptions of management and Safety climate (r = 0.63), but no correlation was exceptionally high.

## Discussion

### Main findings

We investigated the underlying factors and psychometric properties of the Dutch translation of the SAQ-AV in out-of-hours primary care services (GP cooperatives). With exploratory factor analyses, five factors were drawn with reliability scores between .49 and .86: Perceptions of management, Job satisfaction, Teamwork climate, Safety climate and Communication openness. The five-factor model of the Dutch SAQ-AV covered 27 items. Cronbach’s alpha for the factor Communication Openness and the item-total correlations of the items within this factor were low, indicating a problem with the factor. This could be related to the negative wording of all items within this factor which could have had an impact on the variability of the responses (e.g. respondents being reluctant to express negative opinions). However, as the alpha value is influenced by the number of items in a scale [28], the low value of Cronbach’s alpha could also be a consequence of the inclusion of only three items. The factor was not removed from the model based on content considerations. The construct validity was satisfactory for all factors; the moderate correlations of the factors show that there are no two factors measuring the same construct.

### Comparison with other studies

The Norwegian factor structure consisted of five factors covering 30 items, without the factor Communication openness and including the factor Working conditions [19]. The Slovenian structure consisted of the same five factors as the Dutch structure covering 22 items, but with a lot of differences in the items within these factors [6]. At item level, there were more similarities with the Norwegian structure: 17 items fall in the same factors as in Norway whereas 12 items fall in the same factors as in Slovenia. The differences in factor structures between countries make cross-country comparisons of patient safety culture challenging. The structure differences may reflect cross-national variation in the nature and structure of out-of-hours primary care, or mean that item wordings trigger different connotations in the different languages. In a study in the hospital setting, using the Hospital Survey on Patient Safety Culture, more comparable factor structures across countries were found [29].

### Strengths and limitations

The questionnaire was translated using an extensive forward-backward translation procedure and experts checking the relevance of the questions for the Dutch GP cooperative setting. For the factor analysis, we used a large sample of cases. The GP cooperatives were spread across the country and varied in size and degree of urbanisation, contributing to the representativeness of the sample. The participating GP cooperatives together served 13% of the Dutch population.

A limitation of the study is the moderate response rate (43%). We could not perform a non-response bias analysis, but the results indicate that GPs were somewhat underrepresented. Of the respondents, 61% were GPs, but among the invited employees 76% were GPs. Triage nurses were overrepresented—of the respondents 25% were triage nurses, but among the invited employees 16% were triage nurses. This finding is in accordance with the Norwegian study [30].

In order to identify a factor structure it was necessary to remove many items that did not contribute to the measurements. The items may still be valuable in local discussions and interpretations of the results, amongst respondents working on improving their safety culture. But there might also be issues regarding the underlying construction of the total questionnaire. More research into this subject is recommended, for example on how useful these additional items are and whether patient safety culture is better measured with only the items that belong to the factor structure.

## Conclusions

The Dutch translation of the SAQ-AV, with five factors, may be a useful tool for measuring patient safety culture and guide quality improvement interventions in out-of-hours primary care services. It is interesting to gain insight into the factor structures of the SAQ-AV in other countries. Possibilities for comparisons of factor scores across countries seem to be challenging, but comparisons on item level are still an option.

Future studies should examine variation in safety culture between GP cooperatives, and differences in responses between GPs, triage nurses and other professionals. Furthermore, the SAQ should be validated further by examining whether there is an association between patient safety culture, patient experiences, and the occurrence of adverse events.

## Supporting information

S1 File Data file SAQ. (XLS)
